# The effect of feedback on cardiovascular risk factors on optimization of primary prevention: The PharmLines initiative

**DOI:** 10.1016/j.ijchy.2020.100042

**Published:** 2020-07-28

**Authors:** M. Yldau van der Ende, Ingmar E. Waardenburg, E. Lipsic, Jens H.J. Bos, Eelko Hak, H. Snieder, Pim van der Harst

**Affiliations:** a University of Groningen, University Medical Center Groningen, The Department of Cardiology, Groningen, the Netherlands; b PharmacoTherapy, Epidemiology & Economics, Groningen Research Institute of Pharmacy, University of Groningen, Groningen, the Netherlands; c University of Groningen, University Medical Center Groningen, The Department of Epidemiology, Groningen, the Netherlands; d Heart and Lung Division, University Medical Center Utrecht, University of Utrehct, Utrecht, the Netherlands

**Keywords:** Cardiovascular disease, Risk factors, Primary prevention, Lifelines, Risk assessment

## Abstract

**Background:**

It is unknown whether population based single assessment of cardiovascular disease (CVD) risk and feedback to individuals and general practitioners results in initiation of preventive cardiovascular pharmacotherapy in those at risk.

**Methods:**

The population based cohort study Lifelines was linked to the IADB.nl pharmacy database to assess information on the initiation of preventive medication (*N* = 48,770). At the baseline visit, information on cardiovascular risk factors was collected and reported to the participants and their general practitioners. An interrupted-time-series-analysis was plotted, in which the start year of blood pressure and lipid lowering medication was displayed in years before or after the baseline visit. Subsequently, predictors of the initiation of pharmacotherapy were determined and possible reduction in cardiovascular events that could be achieved by optimal treatment of individuals at risk.

**Results:**

Before the Lifelines baseline visit, 34% (out of 1,527, 95% Confidence interval (CI) 32%–36%) and 30% (out of 1,991, 95%CI 28%–32%) of the individuals at risk had a blood pressure or lipid lowering drug prescription, respectively. In those at risk, the use of blood pressure lowering medication, increased substantially during the year of the baseline visit. Treating individuals at increased risk (≥5% 10-year risk) with lipid or blood pressure lowering medication (*N* = 8515 and *N* = 6899) would have prevented 162 and 183 CVD events, respectively, in the upcoming five years.

**Conclusion:**

Primary prevention of CVD in the general population appears suboptimal. Feedback of cardiovascular risk factors resulted in a substantial increase of blood pressure lowering medication and extrapolated health benefits.

## 1 Introduction

In the European Union, cardiovascular death accounts for approximately 37% of total mortality rates [1]. It is thought that a substantial part of cardiovascular mortality can be reduced by early identification and treatment of cardiovascular risk factors [1,2]. Despite numerous studies underlining the importance of early detection [3,4], contradictory evidence is reported on the efficacy of population screening [5,6]. The number of individuals with cardiovascular risk factors is, however, expecting to rise till 2030 [7], driving governments to focus on healthy aging, including cardiovascular disease (CVD) prevention. In the Netherlands, general practitioners (GP) deliver first-line healthcare and have a major role in identification and treatment of individuals at risk for CVD. Identification is usually based on case finding. During a regular visit, the GP decides whether or not to further investigate the presence of cardiovascular risk factors by making inquiries about lifestyle habits and by measuring blood pressure and serum lipid levels. This results in a cardiovascular risk profile, based on the Systematic Coronary Risk Evaluation (SCORE) [8] and a recommendation regarding the start of preventive medication use. Although case finding can be improved via population based studies it remains to be determined whether providing feedback of risk factors via population based studies to participants and GPs results in the initiation of preventive pharmacotherapy. The Lifelines cohort study collects data from 167,729 individuals of the Northern part of the Netherlands [9,10]. During the baseline visit, data on cardiovascular risk factors, including blood pressure and blood lipid levels, is collected and reported to the participants and their GPs. The aim of the current study is to determine the effect of providing feedback on risk factors via a population based study, on initiation of preventive pharmacotherapy.

## 2 Methods

### 2.1 Study design and subjects of the lifelines cohort study

The Lifelines cohort study is a multi-disciplinary prospective population-based cohort study examining in a unique three-generation design the health and health-related behaviors of 167,729 persons living in the North of The Netherlands. It comprises a broad range of investigative procedures in assessing the biomedical, socio-demographic, behavioral, physical and psychological factors which contribute to the health and disease of the general population, with a special focus on multi-morbidity and complex genetics. The study design and rationale of Lifelines were previously described in detail [9]. During the baseline visit an informed consent form was signed, and blood and 24 h urine samples of all participants were collected. Participants underwent a physical examination including anthropometric measurements and a 12-lead electrocardiogram (ECG) [10]. For the current study, all participants without a history of CVD were included. Individuals older than 70 years were excluded since guidelines regarding prevention of CVD focus on individuals aged below 70 years.

### 2.2 Pharmacotherapy

**Fig. 1:**
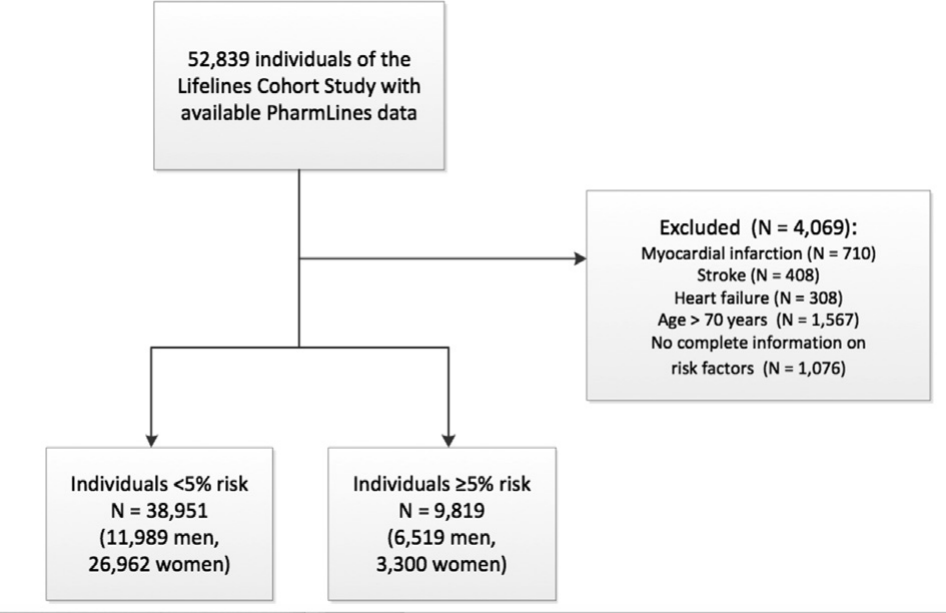
Flowchart of the study population. N = number.

The University of Groningen IADB.nl pharmacy prescription database is a growing database that contains prescription data for more than 20 years from 1996 till 2017 from approximately 70 community pharmacies and covers an estimated population of 700,000 patients [11,12]. Registration in the database is irrespective of health care insurance and age, gender and prescription rates among the database population have been found to be representative of the Netherlands as a whole, and the database has been widely used for research. Each person is individually tracked throughout the database period and prescription records contain information on the date of dispensing, the quantity dispensed, the dose regimen, the number of days the prescription is valid, the prescribing physician and the Anatomical Therapeutic Chemical code (ATC code). Each patient has a unique anonymous identifier; date of birth and gender are known. Due to the high patient-pharmacy commitment in the Netherlands, the medication records for each patient are virtually complete, except for over the counter (OTC) drugs and medication dispensed during hospitalization. Databases from the Lifelines cohort study and IADB.nl were linked by Statistics Netherlands (CBS), which acted as Third Trusted Party. In total, 52,839 individuals of the Lifelines cohort study could be linked to the PharmLines database. We subsequently excluded individuals with previous myocardial infarction (N = 710), stroke (N = 408), heart failure (N = 308), individuals aged > 70 years (N = 1567) and individuals without complete information on cardiovascular risk factors (N = 1,076, Fig. 1).

For each participant, ATC codes and delivery dates were grouped to determine the frequencies of prescriptions of blood pressure and lipid lowering drugs before and after the Lifelines baseline visit in individuals at risk for CVD.

### 2.3 Individuals at risk for cardiovascular disease in the lifelines cohort study

During the Lifelines baseline visit, blood pressure of all participants was measured 10 times. Average blood pressure of the last three measurements was calculated and reported for each participant. Height and weight were measured and used for calculating body mass index (BMI, in kg/m^2^) and serum lipid levels were obtained. Using questionnaires, information about smoking habits, family history of cardiovascular disease and physical activity was collected. Socioeconomic status was defined by highest education level, obtained from questionnaire [13].

After the baseline visit, participants and the GP of each participant received a letter with the individual lab values, anthropometry measures, smoking status and ECG parameters. Abnormal values were highlighted in this letter and included a systolic blood pressure >160 mmHg, a diastolic blood pressure >90 mmHg, BMI <18 kg/m^2^ or BMI >25 kg/m^2^. Abnormal values for lipids included a total cholesterol of >6,5 mmol/L, high density lipoprotein (HDL) ≤1,0 mmol/L, low density lipoprotein (LDL) ≥4,5 mmol/L and triglycerides ≥2,0 mmol/L when this individual was free of CVD and diabetes.

Based on the European Society of Cardiology (ESC) and Nederlands Huisartsen Genootschap (NHG) guidelines it was determined whether preventive medication use was recommended according to each participant's risk profile (Supplementary Table 1). In the ESC guideline, 10-year CVD mortality risk was calculated using the SCORE risk estimate [8,14]. In the NHG guideline, 10-year CVD and CVD mortality risk was calculated based on the SCORE risk, which was recalculated specifically for the Dutch population [15]. Results based on the ESC guideline are published in the main paper and results according to the NHG guideline in the supplementary files.

### 2.4 Statistical analyses

Baseline characteristics of the Lifelines participants who could be linked to the IABD.nl databased are reported. Additionally, baseline characteristics are reported separately for individuals with an estimated 10-year CVD risk <5% and for individuals with an estimated CVD risk ≥5%. Dichotomous variables are presented as frequencies and percentages. Continuous variables are summarized by means and standard deviation (SD) or medians and interquartile ranges (IQRs), as appropriate. The prevalence of blood pressure and lipid lowering drug use was reported as percentages of treated individuals (for men and women separately) of those in whom medication use was recommended according to the ESC or NHG guidelines. Percentages were reported for both medication that was prescribed before the Lifelines baseline visit and initiation of preventive medication after the baseline visit. Additionally, an interrupted time series analyses was plotted, in which the start year of blood pressure and lipid lowering medication was displayed in years before or after the baseline visit. In this analyses data was plotted of individuals in whom medication use was recommended according to the ECS guideline. Controls were defined as individuals in whom no medication use was recommended according to the ECS guideline.

Univariate logistic regression analyses were performed to determine the determinants of pharmacotherapy initiation in individuals eligible for preventive medication (based on either the ESC or NHG guidelines). Initiation of pharmacotherapy before and after the baseline visit were combined in these analyses. Subsequently, a backward-stepwise multiple logistic regression analysis was performed with cutoff for removal set at significance level 0.10 and significance level at 0.05, to determine the independent predictors of this initiation of pharmacotherapy. As sensitivity analyses, a forward-stepwise multiple logistic regression was performed, with cutoff for entry set at a significance level 0.05. P-values <0.05 were considered to be statistically significant.

Subsequently, we calculated the possible 5-year and 3-year CVD reduction that could be gained by treating all individuals with 10-year CVD risk ≥5%, with lipid or blood pressure lowering medication, respectively. In these individuals, 1 mmol/L reduction in LDL cholesterol reduce incident cardiovascular events with 21% in the upcoming 5 years [16]. In a meta-analysis, including 123 studies with a median flow-up time of 3 year (IQR 2–4 years) it was described that 10 mmHg reduction in systolic blood pressure will lead to a reduction of 20% of CVD events and 13% of all-cause mortality [17]. In all individuals with a SCORE risk estimate ≥5% (but without preventive medication use), their risk estimate was multiplied with 0.79 (for reduction of LDL cholesterol on CVD risk), 0.80 (for reduction of systolic blood pressure on CVD risk) and 0.87 (for reduction of systolic blood pressure on mortality risk) respectively, to calculate their individual risk if they would have been treated. Based on these new risk estimates, the absolute numbers of events that could be prevented by treating 1000 individuals were calculated. All statistical analyses were performed using Stata version SE 15.1, StataCorp, College Station, Texas.

## 3 Results

### 3.1 Study population

Baseline characteristics of the remaining 48,770 individuals, as well as separately for individuals with a <5% 10-year CVD risk and individuals >5% 10-year risk, are reported in Table 1. Sixty-two percent (n = 30,262) of these individuals were women and median age was 43 years (IQR 34–50 years).

**Table 1:** Baseline characteristics of individuals of the Lifelines cohort merged with the Pharmlines database.

	Complete population N = 48,770	<5% risk^a^ N = 38,951	≥5% risk^a^ N = 9819	P- value
**Age (median, IQR)**	43 (34–50)	40 (32–46)	53 (60–65)	<0.001
**Female (%*, n*)**	62.1 (30,262)	69.2 (26,962)	33.6 (3300)	<0.001
**SES (%*, n*)**				<0.001
**Low SES**	25.4 (12,109)	21.1 (8015)	43.1 (4094)	
**Middle SES**	36.8 (17,564)	39.3 (15,022)	26.8 (2542)	
**High SES**	37.8 (18,036)	39.7 (15,181)	30.1 (2855)	
**Current smoker (%*, n*)**	21.5 (10,488)	20.5 (7964)	25.7 (2524)	<0.001
**Diabetes mellitus (%*, n*)**	2.9 (1417)	1.9 (733)	7.0 (684)	<0.001
**Anthropometry (mean, SD)**				
Systolic blood pressure (mmHg)	123.5 (14.9)	120.8 (13.2)	134.5 (16.3)	<0.001
Diastolic blood pressure (mmHg)	73.0 (9.3)	71.7 (8.7)	78.1 (9.8)	<0.001
Heart rate	68 (11)	68 (11)	68 (11)	<0.001
**Blood biomarkers (mmol/L; mean, SD)**				
Total cholesterol	5.0 (1.0)	4.9 (0.9)	5.6 (1.1)	<0.001
HDL	1.5 (0.4)	1.5 (0.4)	1.4 (0.4)	<0.001
LDL	3.2 (0.9)	3.1 (0.9)	3.7 (1.0)	<0.001
Triglycerides (median, IQR)	1.0 (0.7–1.4)	0.9 (0.7–1.3)	1.2 (0.9–1.8)	<0.001
**SCORE risk estimate (in %, median, IQR)**	1 (0–1)	0 (0–0)	2 (1–3)	<0.001
**Dutch SCORE risk estimate**^a^**(in %, median, IQR)**	1 (0–4)	1 (0–2)	8 (6–13)	<0.001

a 10-year CVD and CVD mortality risk based on the SCORE risk, but recalculated specifically for the Dutch population. CVD = cardiovascular disease, HDL ratio = high-density lipoprotein, IQR = interquartile range, LDL = low-density lipoprotein, N = number, SCORE = Systematic Coronary Risk Evaluation, SD = standard deviation, SES = socioeconomic status.

### 3.2 Underutilization of preventive pharmacotherapy in patients at increased risk

**Fig. 2:**
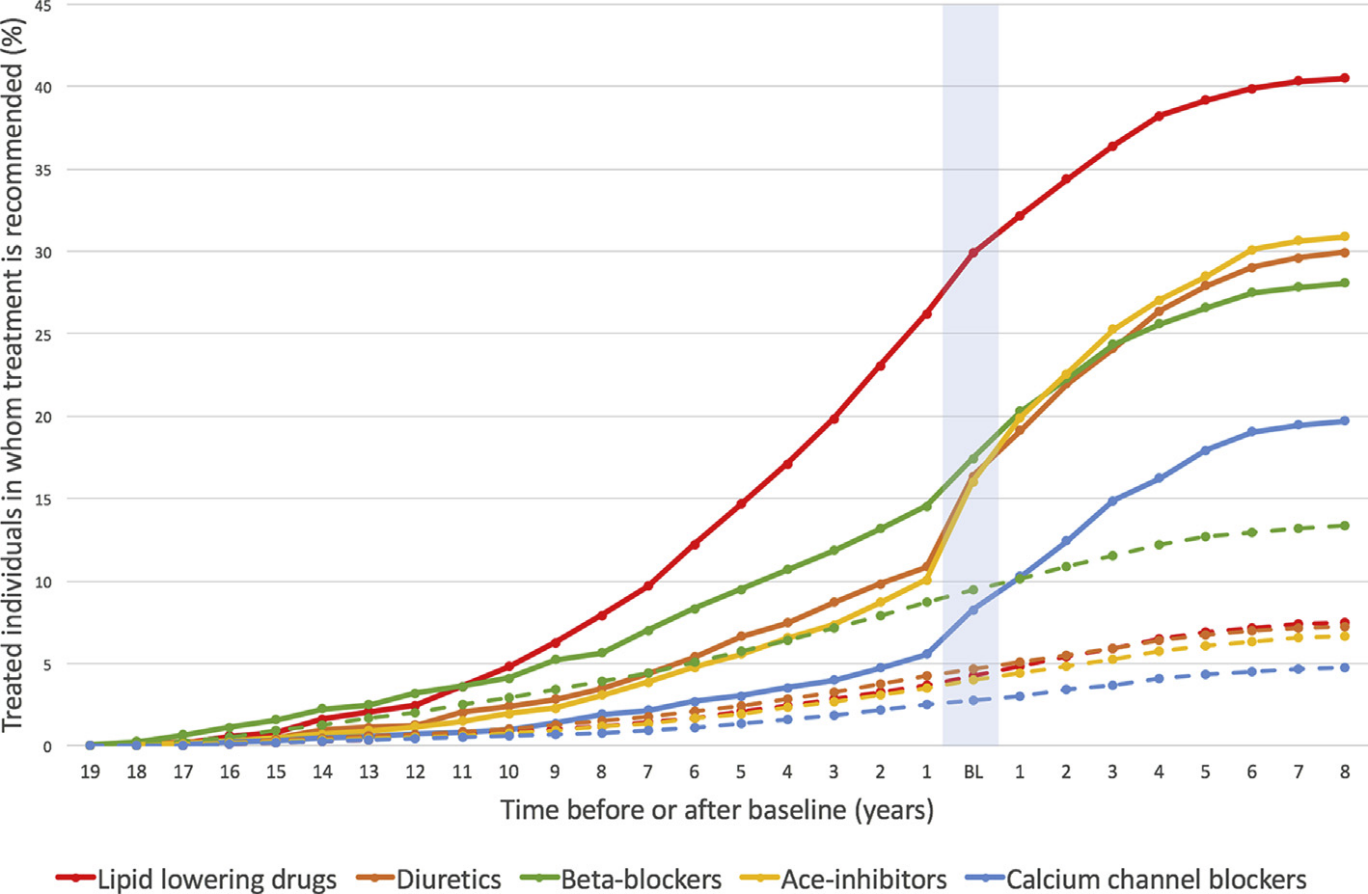
Interrupted time series analysis. On the Y-axis the percentages of treated individuals in whom treatment is recommended and the control group are displayed. On the X-axis the start year of preventive medication is displayed. The light blue vertical line is the year of the baseline visit. **Continues lines** represent the start of medication in individuals at risk. **Dashed lines** represent the start of medication in the control group (individuals without a recommendation for preventive medication). BL = baseline.

According to the European ESC guidelines, 1527 (3.1%) individuals were eligible for blood pressure lowering medication and 1991 (4.1%) for lipid lowering therapy (Supplementary Table 2). Before the baseline visit, treatment percentages among these individuals were 34% (95% Confidence interval (CI) 32%–36%) and 30% (95% CI 28%–32%), respectively. Fig. 2 displays the percentage of treated individuals at risk for CVD as well as the start year of lipid lowering or specific blood pressure lowering medication in individuals at risk and the control group (individuals without a recommendation for preventive medication use). In individuals at risk, the use of blood pressure lowering medication, but not lipid lowering medication, increased substantially during the year of the baseline visit. Among controls, this raise in medication use was not seen.

Women at increased risk more often received blood pressure or lipid lowering medication before the baseline visit compared to men (blood pressure lowering: 42% vs. 30%, P < 0.001, lipid lowering: 37% vs. 25%, P < 0.001, Supplementary Tables 3 and 4). After the baseline visit, initiation of preventive medication use was more similar in men and women. However, a sex difference in initiation of blood pressure lowering medication in the older age category was observed (37% in women aged 50–70 years vs. 30% in men, P = 0.025). Between the two age categories (aged 18–50 years, and 50–70 years), no differences were seen in blood pressure and lipid lowering medication before and after the baseline visit (Supplementary Table 2).

Results according to the Dutch NHG guidelines are reported in Supplementary Tables 2–4, and show similar findings for the initiation of preventive medication after the Lifelines baseline visit.

### 3.3 Predictors of the initiation of pharmacotherapy after single feedback

We determined predictors of the initiation of pharmacotherapy. In univariate logistic regression analyses, female sex, age, socioeconomic status and total cholesterol-HDL ratio were associated with the use of preventive pharmacotherapy in individuals at increased risk according to the ESC guideline. In multivariable logistic regression analyses, female sex and older age remained independent predictors of the initiation of pharmacotherapy (Table 2). Predictors of the start of preventive pharmacotherapy in individuals at high risk according to the NHG guideline are presented in Supplementary Table 5 and include, besides female sex and older age, higher systolic blood pressure and higher total cholesterol-HDL ratio.

**Table 2:** Predictors of prescription of preventive medication before and after baseline visit.

	Univariate logistic regression			Multivariate logistic regression		
	Odds ratio	95% CI	P-value	Odds ratio	95% CI	P-value
Age	1.02	1.01–1.02	<0.001	1.02	1.01–1.03	<0.001
Female	1.32	1.14–1.53	<0.001	1.33	1.13–1.55	<0.001
Low SES *(ref)*						
Middle SES			0.746			
High SES	0.75	0.62–0.920	0.002			
Smoking			0.344			
Systolic blood pressure			0.121			
Diastolic blood pressure			0.663			
TC-HDL ratio	0.94	0.89–1.00	0.040			

Logistic regression analyses on preventive medication prescription before and after baseline in individuals in whom cardio preventive medication is recommended according to the **ESC**guidelines (N = 2930; 1202 women, 1728 men). CI = confidence interval, SES = socioeconomic status, TC-HDL = total cholesterol – high density lipoprotein.

### 3.4 Headroom analysis and possible reduction in cardiovascular events by optimal treatment

Median estimated 10-year cardiovascular risk in individuals with ≥5% cardiovascular event risk, but without lipid lowering medication was 9.0% (N = 8515; IQR 6.0%–18.0%). Decreasing LDL values with 1 mmol/L in those individuals would result in a relative risk reduction of 21% (95% CI 0.19–0.23), leading to a median risk of 7.1% (IQR 4.7%–14.2%). In absolute terms, treating 1000 individuals with a ≥5% cardiovascular event risk with lipid lowering medication would prevent 19 (IQR 13–38) cardiovascular events in the upcoming five years (number needed to treat (NNT): 53).

Median estimated 10-year cardiovascular risk in individuals with ≥5% cardiovascular event risk, but without blood pressure lowering medication was 8.0% (N = 6899; IQR 6.0%–15.0%). Decreasing systolic blood pressure with 10 mmHg in those individuals, would result in a relative risk reduction of 20% (95% CI 0.17–0.23) for CVD events and a reduction of 13% (95% CI 0.09–0.16) in mortality. Treating these individuals would lead to a median risk of 6.4% (IQR 4.8%–12.0%) for CVD events and 7.0% (IQR 5.2%–13.1%) for all-cause mortality. Treating 1000 individuals with blood pressure lowering medication would prevent 16 (IQR 12–30) CVD events (NNT: 63) and 10 (IQR 8–20) deaths (NNT: 100) in three years. Treating all individuals with an increased risk with lipid or blood pressure lowering medication might have prevented 162 and 183 CVD events, respectively, in the upcoming five years.

## 4 Discussion

The main findings of this study are: 1) A substantial part of individuals in the general population are at increased risk for CVD and do not receive preventive pharmacotherapy according to the practice guidelines. 2) The initiation of blood pressure lowering medication among individuals at risk for CVD can likely be increased by a single measurement and feedback to individuals and GPs. 3) Older age and female sex are independent predictors for the initiation of preventive pharmacotherapy. 4) Treating all individuals with increased CVD risk (≥5% 10-year risk) with lipid or blood pressure lowering medication might prevent 162 and 183 CVD events among 48,770 individuals, respectively, in the upcoming five years.

Little real life data is available on preventive cardiovascular treatment of individuals at increased risk in Western Europe. One study investigating the correlation between hypertension treatment and stroke and ischemic heart disease mortality in England, Canada and America reported a strong inverse correlation, with lowest mortality rates in Canada. In Canada, 80% of individuals with hypertension receive medication and hypertension is sufficiently controlled in 66% of these individuals [18]. Indeed, the Canadian government actively encourages patients and caregivers to regularly measure blood pressure and initiate treatment quickly [19]. Another study, using data from 19 countries, including 14 European countries, showed that on average 45% of individuals with hypercholesterolemia use some kind of treatment [20]. In this study is was also determined that the prevalence of drug treatment was strongly correlated with the frequency of screening; supporting the need of screening for cardiovascular risk factors in the general population. The current study shows that one third of the individuals at increased cardiovascular risk of the Lifelines cohort study received adequate preventive pharmacotherapy before the baseline visit in line with practice guidelines. This low proportion of treated individuals suggests that individuals and their doctors might be unaware of their patients risk profile. After a single visit and by providing feedback to individuals and their GP about the risk profiles, preventive mediation use was increased; a finding that suggests that once doctors are aware of their patients risk profile, preventive treatment is likely to be initiated. Interestingly, the use of blood pressure lowering medication, but not lipid lowering medication, increased substantially during the year of the baseline visit. One may speculate that general practitioners are inclined to start with only one type of medication, in combination with lifestyle advise. Blood pressure is an easier measurement, which can be done immediately at first presentation and during follow-up, and may therefore be the first choice to start with.

Through population based cohort studies, like the Lifelines cohort study, there is a possibility to screen for cardiovascular risk factors. Also, governmental screening programs for cardiovascular risk factors may reduce individual burden of CVD and eventually reduce health care costs. In the current study, NNT of 53 and 63 individuals at risk (with a SCORE risk estimate ≥5%) were calculated to prevent one CVD event. So far, contradictory results have been reported regarding the efficacy of population screening [5,6,21] and follow-up studies focused on early detection and treatment of cardiovascular risk factors are needed in terms of outcome and cost-effectiveness.

Older age and female sex are associated with the use of preventive pharmacotherapy in individuals at increased risk. Systematic risk assessment in men younger than 40 years of age and women younger than 50 years of age with no known risk factors is not recommended according to the current guidelines [14]. Older individuals may therefore receive more often preventive medication compared to younger individuals. One might think that women are more likely than men to consult a GP, but studies report that women and men consult their GP just as often [22]. In European countries, cardiovascular risk calculations are based on the SCORE risk estimate [8]. It has been described that the estimated 10-year cardiovascular mortality calculated by SCORE, seriously underestimates overall cardiovascular risk, especially in women and younger individuals [23]. Women and younger individuals have higher levels of for example blood pressure or lipid levels, before treatment is recommended as compared to men, which might be an explanation of the higher prevalence of treatment among women with high estimated CVD risk. Given the underestimation of the SCORE risk estimate; the number of undertreatment of individuals at risk may even be higher than reported.

### 4.1 Limitations

The study has several limitations. First, the IADB.nl database only includes individuals who ever had a drug prescription. Lifelines participants without any prescriptions could therefore not be linked with the IADB.nl database. Thus, the number of individuals at risk without preventive medication use can even be higher than reported in the current study. Also, the IADB.nl database does not cover the entire population of the Northern provinces of the Netherlands yet. In future, when more data is collected, more precise estimates on the use of cardiovascular preventive medication could be made. Nevertheless, the current linked database, including 50,000 individuals, is the largest contemporary database to examine CVD prevention in the Netherlands. Third, we were not able to establish possible explanations for the undertreatment of cardiovascular risk factors, such as patient factors (e.g. medication intolerance or poor compliance) or doctor factors (e.g. absence of programmatic cardiovascular risk management). Finally, although treatment numbers increased after the Lifelines baseline visit, we are not able to draw any causal conclusions because of potential unmeasured confounding.

## 5 Conclusion

Primary prevention of CVD is suboptimal in the general population possibly by unawareness of risk factors. Providing feedback on the presence of cardiovascular risk factor by population based measurements likely results in an increase in blood pressure lowering medication use. Optimal treatment of individuals at risk for CVD may have the potential to reduce the burden of CVD events.

## Sources of funding

The Lifelines Biobank initiative has been made possible by funds from FES (Fonds Economische Structuurversterking), 10.13039/501100013427SNN (Samenwerkingsverband Noord Nederland) and REP (Ruimtelijk Economisch Programma). The IADB.nl is funded by the 10.13039/501100001721University of Groningen.

## Author statement

**Van der Ende MY:** Conceptualization, Methodology, Software, Writing- Original draft preparation.

**Waardenburg I:** Writing- Original draft preparation.

**Lipsic E:** Supervision, Writing- Reviewing and Editing.

**Bos JHJ:** Software, Validation, Reviewing, Data Curation.

**Hak E:** Software, Validation, Reviewing.

**Snieder H:** Supervision, Writing- Reviewing and Editing.

**Van der Harst P:** Supervision, Writing- Reviewing and Editing.

## Declaration of competing interest

The authors declare that they have no known competing financial interests or personal relationships that could have appeared to influence the work reported in this paper.
