# Supplementary material for: The effect of feedback on cardiovascular risk factors on optimization of primary prevention: The PharmLines initiative
Source: Int J Cardiol Hypertens. 2020 Jul 28;6:100042. doi: 10.1016/j.ijchy.2020.100042 (PMC7803074; doi:10.1016/j.ijchy.2020.100042)
Supplement: Multimedia component [file mmc1.docx]

**Supplementary files**

**Supplementary Table 1.**

Recommendations for preventive medication use according to the ESC and NHG guidelines.

| **Guideline** | **Recommendation preventive blood pressure lowering or lipid lowering medication in individuals (free of CVD) with:** |
| --- | --- |
| **ESC** | **1)** SCORE risk estimate ≥10%, with a LDL>1.8 mmol/L or a SBP or DBP ≥140 and/or ≥90 mmHg.  **2)** SCORE risk ≥5% and <10**%** with a LDL >2.6 mmol/L.  **3)** SBP ≥180 mmHg or a DBP ≥110 mmHg irrespective of their cardiovascular risk estimate.  **4)** Lipid lowering medication is recommended in all individuals with diabetes mellitus aged 40 years or older. |
| **NHG** | **1)** 10-year risk estimate on CVD ≥ 20%, with a SBP>140 mmHg and/or LDL>2.5 mmol/L  **2)** 10-year risk estimate on CVD of 10-20%, with SBP>140 mmHg and/or LDL>2.5 mmol/L ***and*** the presence of either family risk of CVD, inactivity or obesity  **3)** isolated SBP>180 mmHg or TC/HDL-ratio>8, irrespective of other risk factors. |

CVD = cardiovascular disease, DBP = diastolic blood pressure, ESC = European Society of Cardiology, LDL = low-density lipoprotein, NHG = Nederlands Huisartsen Genootschap, SCORE = Systematic Coronary Risk Evaluation, SPB = systolic blood pressure, TC/HDL ratio = total cholesterol/high-density lipoprotein ratio

**Supplementary Table 2.**

Prescriptions of blood pressure lowering and lipid lowering medication in individuals at risk for cardiovascular disease according to the ESC and NHG guidelines.

|  | **AGE 18-50** | **AGE 50-70** |
| --- | --- | --- |
| **ESC: Blood pressure lowering medication recommended** | ***n* = 496** | ***n* = 1,031** |
| *Blood pressure lowering at baseline (%, n)* | 32.7 (162) | 34.7 (358) |
| *Blood pressure lowering after baseline (%, n)* | 34.9 (173) | 32.5 (335) |
| Total *(%, n)* | 67.6 (335) | 67.2 (693) |
| **ESC: Lipid lowering medication recommended** | ***n* = 400** | ***n* = 1,591** |
| *Lipid lowering at baseline (%, n)* | 30.25 (121) | 29.79 (474) |
| *Lipid lowering after baseline (%, n)* | 11.0 (44) | 10.56 (168) |
| Total *(%, n)* | 41.25 (165) | 40.35 (642) |
| **NHG: Blood pressure lowering medication recommended** | ***n* = 51** | ***n* = 675** |
| *Blood pressure lowering at baseline (%, n)* | 39.2 (20) | 37.78 (255) |
| *Blood pressure lowering after baseline (%, n)* | 37.3 (19) | 32.2 (217) |
| Total *(%, n)* | 76.5 (39) | 69.98 (472) |
| **NHG: Lipid lowering medication recommended** | ***n* = 149** | ***n* = 1,087** |
| *Lipid lowering at baseline (%, n)* | 7.38 (11) | 14.90 (162) |
| *Lipid lowering after baseline (%, n)* | 15.44 (23) | 14.08 (153) |
| Total *(%, n)* | 22.82 (34) | 28.98 (315) |

**Supplementary Table 3.**

Prescriptions of blood pressure lowering and lipid lowering medication in **women** at risk for cardiovascular disease according to the ESC and NHG guidelines.

|  | **AGE 18-50** | **AGE 50-70** |
| --- | --- | --- |
| **ESC: Blood pressure lowering medication recommended** | ***n* = 213** | ***n* = 348** |
| *Blood pressure lowering at baseline (%, n)* | 42.2 (90) | 41.1 (143) |
| *Blood pressure lowering after baseline (%, n)* | 36.2 (77) | 37.1 (129) |
| Total *(%, n)* | 78.4 (167) | 78.2 (272) |
| **ESC: Lipid lowering medication recommended** | ***n* = 210** | ***n* = 527** |
| *Lipid lowering at baseline (%, n)* | 27.6 (58) | 41.4 (218) |
| *Lipid lowering after baseline (%, n)* | 9.1 (19) | 8.5 (45) |
| Total *(%, n)* | 36.7 (77) | 49.9 (263) |
| **NHG: Blood pressure lowering medication recommended** | ***n* = 22** | ***n* = 225** |
| *Blood pressure lowering at baseline (%, n)* | 54.6 (12) | 44.4 (100) |
| *Blood pressure lowering after baseline (%, n)* | 40.9 (9) | 32.9 (74) |
| Total *(%, n)* | 95.5 (21) | 77.3 (174) |
| **NHG: Lipid lowering medication recommended** | ***n* = 28** | ***n* = 353** |
| *Lipid lowering at baseline (%, n)* | 14.3 (4) | 16.4 (58) |
| *Lipid lowering after baseline (%, n)* | 10.7 (3) | 13.3 (47) |
| Total *(%, n)* | 25.0 (7) | 29.7 (105) |

**Supplementary Table 4.**

Prescriptions of blood pressure lowering and lipid lowering medication in **men** at risk for cardiovascular disease according to the ESC and NHG guidelines.

|  | **AGE 18-50** | **AGE 50-70** |
| --- | --- | --- |
| **ESC: Blood pressure lowering medication recommended** | ***n* = 283** | ***n* = 683** |
| *Blood pressure lowering at baseline (%, n)* | 25.4 (72) | 31.5 (215) |
| *Blood pressure lowering after baseline (%, n)* | 33.9 (96) | 30.2 (206) |
| Total *(%, n)* | 59.4 (168) | 61.6 (421) |
| **ESC: Lipid lowering medication recommended** | ***n* = 190** | ***n* = 1,064** |
| *Lipid lowering at baseline (%, n)* | 33.2 (63) | 24.1 (256) |
| *Lipid lowering after baseline (%, n)* | 13.2 (25) | 11.6 (123) |
| Total *(%, n)* | 46.3 (88) | 35.6 (379) |
| **NHG: Blood pressure lowering medication recommended** | ***n* = 29** | ***n* = 450** |
| *Blood pressure lowering at baseline (%, n)* | 27.6 (8) | 34.4 (155) |
| *Blood pressure lowering after baseline (%, n)* | 34.5 (10) | 31.8 (143) |
| Total *(%, n)* | 62.1 (18) | 66.2 (298) |
| **NHG: Lipid lowering medication recommended** | ***n* = 121** | ***n* = 734** |
| *Lipid lowering at baseline (%, n)* | 5.8 (7) | 14.2 (104) |
| *Lipid lowering after baseline (%, n)* | 16.5 (20) | 14.4 (106) |
| Total *(%, n)* | 22.3 (27) | 28.6 (210) |

**Supplementary Table 5.**

Predictors of prescription of preventive medication before and after the baseline visit.

Logistic regression analyses on preventive medication prescription before and after baseline in individuals in whom cardio preventive medication is recommended according to the **NHG** guidelines (N = 1,369; 446 women, 923 men)

|  | **Univariate logistic regression** | | | **Multivariate logistic regression** | | |
| --- | --- | --- | --- | --- | --- | --- |
|  | **Odds ratio** | **95% CI** | **P-value** | **Odds ratio** | **95% CI** | **P-value** |
| Age | 1.03 | 1.02 – 1.04 | <0.001 | 1.04 | 1.03 – 1.06 | <0.001 |
| Female | 1.39 | 1.11 – 1.75 | 0.004 | 1.27 | 1.00 – 1.62 | 0.048 |
| Low SES *(ref)* |  |  |  |  |  |  |
| Middle SES | 0.77 | 0.59 – 1.01 | 0.056 |  |  |  |
| High SES | 0.74 | 0.57 – 0.96 | 0.023 |  |  |  |
| Smoking |  |  | 0.771 |  |  |  |
| Systolic blood pressure | 1.02 | 1.01 – 1.02 | <0.001 | 1.02 | 1.01 – 1.02 | <0.001 |
| Diastolic blood pressure | 1.01 | 1.00 – 1.02 | 0.012 |  |  |  |
| TC-HDL ratio | 0.90 | 0.85 – 0.94 | <0.001 | 1.08 | 1.01 – 1.17 | 0.035 |

TC-HDL = total cholesterol – high density lipoprotein
